# Genome-Wide Identification and Characterization of *Fusarium graminearum*-Responsive lncRNAs in *Triticum aestivum*

**DOI:** 10.3390/genes11101135

**Published:** 2020-09-27

**Authors:** Xiaoxin Duan, Xiushi Song, Jianxin Wang, Mingguo Zhou

**Affiliations:** 1 Laboratory of Plant Disease Control and Phytopharmacy, College of Plant Protection, Nanjing Agricultural University, Nanjing 210095, China; 2017202047@njau.edu.cn (X.D.); songxiushi_01@163.com (X.S.); jlz489@163.com (J.W.); 2 The Key Laboratory of Plant Immunity, Nanjing Agricultural University, Nanjing 210095, China

**Keywords:** wheat, *Fusarium graminearum*, lncRNA, identification, meta-QTL

## Abstract

Although the war between wheat and *Fusarium* has been widely investigated for years, long noncoding RNAs (lncRNAs), which have been proven to regulate important processes in the development and stress responses of plants, are still poorly known in wheat against *Fusarium.* Herein, we systematically reveal the roles of wheat lncRNAs in the process of *Fusarium graminearum* infection by high-throughput RNA sequencing. Well over 4130 of the total 4276 differentially expressed lncRNAs were already specifically expressed at 12 h postinoculation (hpi), but only 89 of these were specifically expressed at 24 hpi, indicating that the initial stage was the crucial stage for lncRNA-mediated gene regulation of wheat defense against *F. graminearum*. Target analysis showed the lncRNAs participated in various biological stress processes and had exclusive regulation models at different infection stages. Further H_2_O_2_ accumulation and protein ubiquitination assays supported this idea. Moreover, two lncRNAs (XLOC_302848 and XLOC_321638) were identified as Fusarium seedling blight resistance candidates by lncRNA-target expression pattern validation, and two lncRNAs (XLOC_113815, XLOC_123624) were Fusarium head blight resistance potential regulators by cross-validating the RNAseq data with the refined meta-QTL of wheat FHB resistance. These findings extend our knowledge on wheat lncRNAs response to *F. graminearum* attack and provide new insights for the functional and molecular research of future interactions between wheat and *Fusarium*.

## 1. Introduction

Over the years, protein-coding genes have been given the attention of researchers, while with improvements in high-throughput sequencing technology, people have found that about 90% of the genome of an organism is transcribed into RNAs, the majority of them noncoding [1]. Based on their length, noncoding RNAs are arbitrarily grouped into small RNAs (< 200 nt) and long noncoding RNAs (lncRNAs, longer than 200 nt) [2,3]. Studies in animals and humans have shown that lncRNAs play a crucial role in regulating various biological processes, such as transcriptional regulation, genomic imprinting, and dosage compensation [4,5,6]. Plants have fewer lncRNA than animals, while these RNAs perform an almost-similar function in plants. To date, amounts of lncRNAs have been identified in *Arabidopsis* [7], wheat [8], rice [9], maize [10], cotton [11], and populus [12]. Some of them have been proven to be functionally characterized. For example, two lncRNAs from *Arabidopsis*, *COOLAIR* and *COLDAIR*, have been characterized from FLOWERING LOCUS C (FLC), which acts as a floral repressor [13,14]. In cotton, GhlncNAT-ANX2 and GhlncNAT-RLP7 displayed an enhanced resistance towards *Verticillium dahliae* and *Botrytis cinerea* when they were silenced [15]. Three phosphate-deficiency-induced lncRNAs *PDILs* could mediate the signaling of Pi deficiency and Pi transport, suggesting that lncRNAs were involved in the plant responses to Pi deficiency [16]. These findings imply that plant lncRNAs take on significant functions in growth, development, and various biotic or abiotic stress responses. 

Wheat (*Triticum aestivum*) is one of the most extensively grown and vital food crops for humans due to its abundant nutrition and wide adaptability to the environment. However, the disease epidemics in wheat caused by *Fusarium* are frequently recorded in the United States, the United Kingdom, and China, which are becoming majorly threatened by economic damage related to crop protection [17,18]. To make matters worse, *Fusarium* pathogens produce various types of mycotoxins that are immunotoxic and carcinogenic and are thus highly detrimental to human and animal health [19,20]. The most severe diseases caused by *Fusarium sp*. include Fusarium head blight (FHB) and Fusarium seedling blight (FSB) in wheat, and *Fusarium graminearum* is a major pathogen among *Fusarium sp.* [21]. In the evolution of plants, the common signaling networks governing the defense responses, such as the antagonistic signaling pathways of jasmonic and salicylic acids, multiple independent expansions of resistance genes, and resistance gene-regulating microRNAs, have also undergone complex evolution to cope with pathogen attacks [22]. In wheat, a large number of genes that function in FHB resistance have been identified, including *Fhb1*, plant defensin 1.2 (*pdf1.2*), chitinase-1 (*Chi*-1), glucanase-2 (*Glu*-*2*), uridine diphosphate-glucosyltransferase (*UGTs*), *TaWRKY45,* and so on [23,24,25,26,27,28]. Contrasted with protein-coding genes, defense-response-related lncRNAs are less well documented in wheat immunity. A recent study shows that numerous lncRNAs were expressed in wheat to defend the infection of *Puccinia striiformis* and *Blumeria graminis*, including 254 long intergenic noncoding RNAs (lincRNAs) responding to *B. graminis* stress and 52 lincRNAs responding to *P. striiformis*; meanwhile, 101 differentially expressed lincRNAs were predicted as targets of miRNA and 5 were target mimics [29].

Here, we identify lncRNAs and analyze their function in *T. aestivum* against *Fusarium graminearum* for the first time. We constructed a completed lncRNA profile that contains the expressional information of 126,391 high-confidence lncRNAs in *T. aestivum* defending *F. graminearum*. Among those lncRNAs, 4276 lncRNAs were identified as differentially expressed under *F. graminearum* attack, and 96.6% of them were specifically expressed at 12 h postinoculation (hpi), suggesting that the lncRNAs played an important role in defending *F. graminearum* infection at the initial stage. To further analyze the regulation model of lncRNAs at different stages, we predicted the target genes of lncRNA and classified their functional categories by GO and KEGG enrichment analysis. It showed that stress-stimulus-related genes were only found in wheat at 12 hpi, while peptidase-related genes were specifically enriched in wheat 24 hpi. In addition, H_2_O_2_ accumulation and protein ubiquitination assays verified the above results. Finally, two lncRNAs, XLOC_302848 and XLOC_321638, were identified as FSB-resistance candidates by qPCR-mediated lncRNA-target expression pattern validation. Additionally, two lncRNAs, XLOC_113815 and XLOC_123624, were identified as FHB-resistance potential regulators by cross-validating the RNAseq data with the refined meta-QTL analysis. Our results provide candidates that can be used to improve wheat resistance against *Fusarium* and extend our knowledge on plant lncRNAs that regulated plant response.

## 2. Materials and Methods 

### 2.1. Plant Materials and Fungal Pathogen Inoculation 

The *T. aestivum* cv. LiangXing 66 was used throughout all experiments. *T. aestivum* seeds were grown in a light incubator at 25 °C for two and a half days, under a photoperiod of 16 h light and 8 h dark, then the tips of the coleoptile were cut off quickly with aseptic scissors for *F. graminearum* inoculation. A 3-acetyldeoxy-nivalenol (3ADON) chemotype strain *F. graminearum* 2021 [30,31], isolated from a field experiencing an FHB epidemic in Zhejiang Province, China, was used as the parental strain in this study. *F. graminearum* 2021 were cultivated on potato dextrose agar (PDA) medium for 3–4 days, and then, high-activity hyphae at the edge of the colony were collected and shook in 3% sterile mung bean broth at 25 °C at 175 rpm for 3 days. The conidia were collected and suspended with deionized water at a concentration of 1.0 × 10^6^ spores/mL.

Treated coleoptiles were inoculated with 3 µL of conidia suspension (1.0 × 10^6^ spores/mL) and then returned into the incubator. After 12 and 24 hpi, the coleoptile inoculation sites, about 3 mm, were harvested. At the same time, coleoptiles treated with distilled water were collected as mock treatments. Once harvested, all samples were frozen in liquid nitrogen as quickly as possible and stored at −80 °C for RNA-Seq.

### 2.2. RNA Isolation, Library Construction, and Sequencing

High-quality RNA was isolated from the samples using the Total RNA Kit (Tiangen, Beijing, China). Then, 1% agarose gel was used to detect RNA degradation and contamination. Libraries were constructed using the Illumina rRNA-depleted RNA by NEBNext Ultra™ Directional RNA Library Prep Kit (NEB, USA), following the manufacturer’s recommendations. Strand-specific sequencing was performed on the Illumina Hiseq 4000 platform (paired-end 150 base pair reads).

### 2.3. LncRNA Identification 

After sequencing, all data were processed by removing adaptors and low-quality reads (the ration of the base with Q < 5 should be > 50% of the whole read) using FastQC [32]. Then, we mapped those clean reads independently to the *T. aestivum* reference genome from the website (ftp://ftp.ensemblgenomes.org/pub/plants/release-41/fasta/triticum_aestivum) using HISAT2 v2.0.4 [33]. Each transcriptome was assembled and separated by StringTie v1.3.1 in a reference-based approach [34]. Before identification, we used the Cuffmerge [35] to combine the transcripts obtained by splicing each sample and removed the transcript whose chain direction was uncertain, so as to obtain the complete transcript of this sequencing.

The following five steps were applied to identify lncRNAs from the transcriptome assemblies: (1) Select transcripts with exon number ≥ 1 and filter out numerous single-exon transcripts by FPKM (fragments per kilobase of exon per million fragments mapped) value < 0.5 through Cuffquant [36], (2) select transcripts that have a length of more than 200 bp; (3) filter out transcripts overlapped with the exon region of the database annotation by Cuffcompare software, and include the lncRNAs overlapping the exon region of the spliced transcript in the database as database annotation lncRNAs for subsequent analysis, (4) retain the transcripts characterized by FPKM value ≥ 0.5 (for single-exon transcripts, FPKM value ≥ 2), and (5) remove the transcripts that fail to pass the protein-coding-score test using the coding–noncoding index (CNCI) [37], the coding-potential calculator (CPC) [38], and Pfamscan [39] software. 

### 2.4. Analysis of Differential Expression Patterns

We employed Cuffdiff v2.1.1 software to calculate the FPKM values of both lncRNAs and coding genes in each sample to examine the gene expression levels, and genes expressed differentially were identified with an adjusted *p*-value < 0.05 [40]. The expression of lncRNAs was normalized and then clustered into several groups by hierarchical clustering [41].

### 2.5. GO and KEGG Enrichment Analysis

Given that lncRNA can regulate the expression of its neighboring genes at the transcriptional level [5], we analyzed the genomic colocations between lncRNA and their neighboring genes. We defined the gene within 100 kb upstream and downstream of each lncRNA as the putative target gene (PT gene) [42]. The relative loci between lncRNAs and their PT genes can be exhibited using Integrative Genomics Viewer (IGV) software (http://www.broadinstitute.org/igv/) [43]. Function annotation of PT genes colocated with the differentially expressed lncRNAs was conducted with Gene Ontology (GO) and Kyoto Encyclopedia of Genes and Genomes (KEGG) analyses. GO enrichment analysis was performed using the GOseq R package, and GO terms with corrected *p*-values less than 0.05 were considered significantly enriched [44]. KEGG-orthology-based annotation system (KOBAS) software was used to test for the statistical significance of the differentially expressed lncRNA PT genes in KEGG pathways (http://www.genome.jp/kegg) [45,46].

### 2.6. H_2_O_2_ Accumulation and Ubiquitination Detection Assays

3,3-Diaminobenzidine (DAB) staining and Western blotting assays were used to detect the levels of reactive oxygen species (ROS) and protein ubiquitination, respectively. Coleoptiles after 12 and 24 hpi were prepared for staining and protein extraction. For DAB staining, samples were incubated in a DAB solution (1 mg/mL) for 10 h at 28 °C in the dark and then decolorized with 95% ethanol at 70 °C for 30 min. For Western blotting, about 250 mg liquid nitrogen ground coleoptile was resuspended in 1 mL of RIPA lysate (1 M Tris-HCL, PH 8.0, 150 mM NaCl, 1% Triton X-100, 0.1% SDS), with 10 µL protease inhibitor cocktail (Sangon, Shanghai, China) to extract the total protein. After 1 h of incubation on ice, homogenizing with a vortex shaker every 10 min, the lysis solution was centrifuged at 12,000× g in a microcentrifuge for 15 min at 4 °C. Samples with consistent protein concentration were loaded onto 10% SDS-PAGE gels, respectively. Proteins separated on gels were transferred to an Immobilon-P transfer membrane (Millipore, Billerica, MA, USA). Ubiquitination signals were detected by a rabbit antiubiquitin antibody (382766, Zenbio, Chengdu, China) at 1:2000 dilution, and a monoclonal anti-β-actin antibody (700068, Zenbio) was used as a reference. Secondary antibody incubation and chemiluminescent detection were performed as previously described [47]. Gray value analysis was performed using ImageJ 1.53a software (National Institutes of Health, Bethesda, MD, USA).

### 2.7. Quantitative Real-Time (RT) PCR and LncRNA-PT Genes Network Analysis

Fourteen lncRNAs from six stress response GO terms (Table S1) responding to *F. graminearum* attack were selected to evaluate the defense expression profiles and 4 pairs of lncRNA-PT genes were used to verify the defense expression patterns. RNA samples were extracted with a Total RNA Kit (Tiangen) from wheat coleoptile inoculation sites grown for 6, 12, 24, 36, and 48 hpi. About 1000 ng RNA in a volume of 20 µL was reverse-transcribed into first-strand cDNA using a PrimeScript RT reagent kit (TaKaRa), according to the manufacturer’s protocol. Quantitative RT-PCR (qRT-PCR) was performed in a 20 µL reaction volume containing primers, ChamQ SYBR qPCR Master Mix (Vazyme, Nanjing, China), and diluted cDNA templates on the ABI 7500 real-time detection system (Applied Biosystems, Foster City, CA, USA). The primers used for qRT-PCR analysis are listed in Table S2. Sample cycle threshold (CT) values were determined and standardized relative to the endogenous control actin gene (Gene-Bank: AB181991.1), and the 2^–ΔΔCT^ method was used to calculate the relative fold changes of gene expression. For the lncRNA-PT genes network, we firstly cross-validated the differentially expressed protein-coding genes of our transcriptional data with the genes of meta-QTL 1/Chr. 3B, which were found to be the best QTL loci to mine candidate genes [48], and then screened the cross-validated genes neighboring one or more lncRNAs identified in our study. A lncRNA-PT genes network was built by using *Cytoscape* software.

## 3. Results

### 3.1. Genome-Wide Identification and Characterization of lncRNAs in Wheat

**Figure 1:**
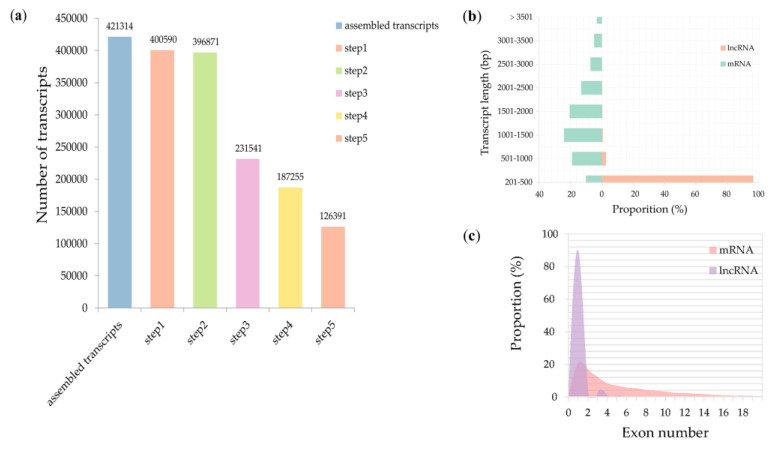
Identification and characterization of long noncoding RNAs (lncRNAs). (**a**) Statistics of candidate lncRNAs. Step 1: select transcripts with exon number ≥ 1 and filter out numerous single-exon transcripts by FPKM (fragments per kilobase of exon per million fragments mapped) value < 0.5. Step 2: select transcripts with length > 200 bp. Step 3: filter out transcripts overlapped with the exon region of the database annotation. Step 4: retain the transcripts characterized by FPKM value ≥ 0.5 (for single-exon transcripts, FPKM value ≥ 2). Step 5: remove the transcripts that failed to pass the protein-coding-score test. (**b**) Comparison of the transcript length between lncRNAs and mRNAs; (**c**) exon number distribution of lncRNAs and mRNAs.

Two time-points, 12 and 24 h, were selected to collect coleoptiles for sequencing. We generated eight high-depth transcriptomes. The four of them were generated from coleoptiles inoculated with water, and the others were generated from coleoptiles inoculated with *F. graminearum*. In total, more than 786 million clean reads were obtained from the Illumina Hiseq 4000 system. An integrated approach (see Materials and Methods) was further used to identify high-confidence lncRNAs. We discovered 421,314 total transcripts from assembled RNA seq data and subjected them to five sequential stringent filter processes to extract lncRNA transcripts (Figure 1a). About 70% (294,923) transcripts were discarded due to their length being shorter than 200 bp, FPKM < 0.5, or not passing the protein-coding-score test. We ultimately identified 126,391 transcripts as high-confidence lncRNAs in *T. aestivum* against *F. graminearum*. Three categories of lncRNAs were classified, and lincRNAs were the majority of lncRNAs, followed by antisense lncRNAs and intronic lncRNAs (Table 1). To compare the character differences between putative lncRNAs identified in this study and mRNAs, we explored their length and exon distribution. We found that the majority of lncRNAs (96.3%) were 201–500 bp in length, while the majority of mRNAs (62.7%) were 500–2000 bp in length (Figure 1b, Table S3). Analysis of exon number revealed that 90% lncRNAs contained single-exonic transcripts, while only 20.6% mRNAs contained single-exonic transcripts (Figure 1c). 

### 3.2. Massive Differentially Expressed lncRNAs Responding to F. graminearum Were Identified at the Initial Stage Rather Than Invasive Stage

**Figure 2:**
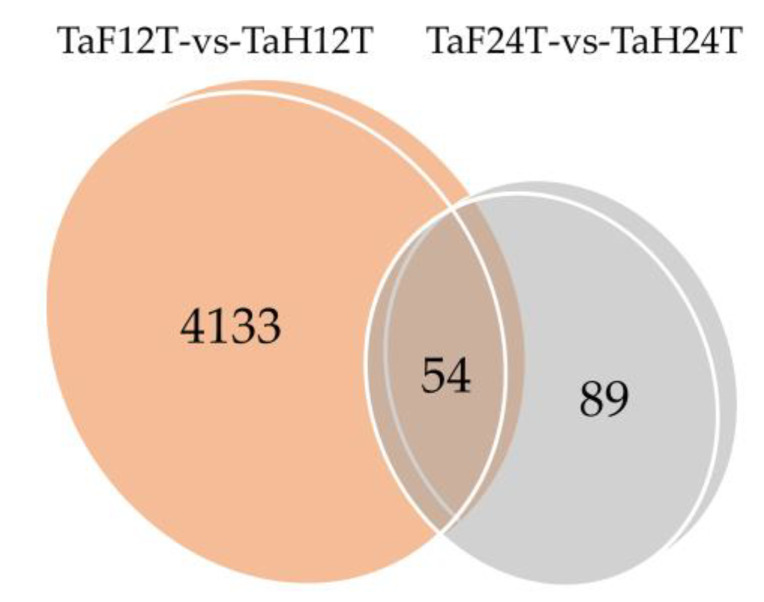
Venn diagram of 12 and 24 h common/specific differentially expressed lncRNAs. TaF12T, samples at 12 h inoculation with *F. graminearum*; TaH12T, samples at 12 h inoculation with distilled water; TaF24T, samples at 24 h inoculation with *F. graminearum*; TaH24T, samples at 24 h inoculation with distilled water. TaF12T-vs-TaH12T, differentially expressed wheat lncRNAs after 12 h inoculation with *F. graminearum*. TaF24T-vs-TaH24T, differentially expressed wheat lncRNAs after 24 h inoculation with *F. graminearum*.

**Figure 3:**
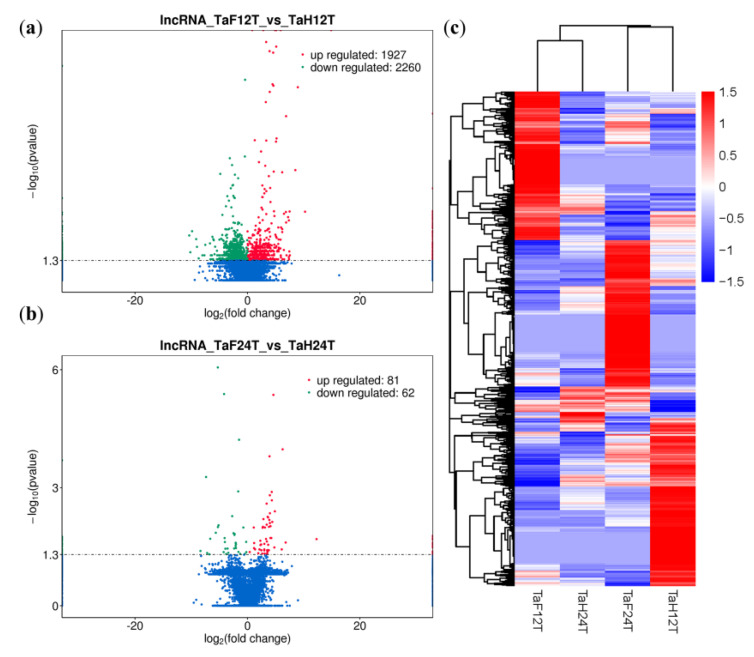
Analysis of differentially expressed lncRNAs (DE lncRNAs) of wheat infected by *F. graminearum*. (**a**) Volcano plot of DE lncRNAs at 12 hpi. lncRNA_TaF12T_vs_TaH12T, wheat DE lncRNAs after 12 h inoculation with *F. graminearum*. The red and green points represent upregulated and downregulated lncRNAs, respectively. (**b**) Volcano plot of DE lncRNAs at 24 hpi. lncRNA_TaF24T_vs_TaH24T, wheat DE lncRNAs after 24 h inoculation with *F. graminearum*. (**c**) Hierarchical clustering plot of DE lncRNAs. TaF12T, samples at 12 h inoculation with *F. graminearum*; TaH12T, samples at 12 h inoculation with distilled water; TaF24T, samples at 24 h inoculation with *F. graminearum*; TaH24T, samples at 24 h inoculation with distilled water. Different columns represent different samples, and different rows represent different genes. Data are expressed as FPKM. Red: relatively high expression; Blue: relatively low expression.

To illuminate the expression changes of lncRNAs at different stages under *F. graminearum* attack, the expression profile was constructed by comparing the normalized expression FPKM (*p*-value < 0.05, log2 fold change ≥ 1). We identified 4276 lncRNAs that were differentially expressed under *F. graminearum* attack, 4133 of which were specifically expressed at 12 hpi and 89 at 24 hpi (Figure 2, Table S4), suggesting that the initial stage (12 hpi) rather than the invasive stage (24 hpi) may be a crucial stage for lncRNA-mediated gene regulation of wheat defending an *F. graminearum* infection. It was worth noting that 54 lncRNAs were codifferentially expressed despite the expression value being varied at 12 and 24 hpi. The number of downregulated lncRNAs was 17% higher than that of upregulated lncRNAs at 12 hpi, whereas 33% were lower at 24 hpi (Figure 3a,b). Total differentially expressed lncRNAs (DE lncRNAs) were clustered in a heat map, which represents the relative expression pattern of 4276 lncRNAs of *T. aestivum* in two-time stages after *F. graminearum* attack. As shown in Figure 3c, the expression pattern of lncRNAs in wheat inoculated with *F. graminearum* was clearly separated from that inoculated with distilled water. One lncRNA cluster was upregulated in wheat 12 hpi with *F. graminearum* but was downregulated in wheat 12 hpi with water and wheat 24 hpi with both *F. graminearum* and water (Figure 3c, top panel). Another cluster was upregulated in wheat 24 hpi with *F. graminearum* but was downregulated in other treatments (Figure 3c, middle panel). These results indicated that lncRNAs were significantly induced in wheat defense responses.

Interestingly, after *F. graminearum* inoculation, the expressional pattern of mRNA was similar to that of lncRNA. Approximately 11,307 and 971 mRNAs were unique at 12 and 24 hpi, respectively, and 2371 mRNAs were codifferentially expressed (data not shown). In addition, by using the Circos program [49], these differentially expressed lncRNAs and mRNAs were both evenly distributed across 15 chromosomes of wheat and showed no obvious preferences of locations, indicating that multiple lncRNAs and mRNAs participated in wheat defense and formed a complicated network together (Figure S1).

### 3.3. Targets Analysis Revealed F. graminearum-Responsive lncRNAs Have Their Own Exclusive Functional Models at Different Stages

**Figure 4:**
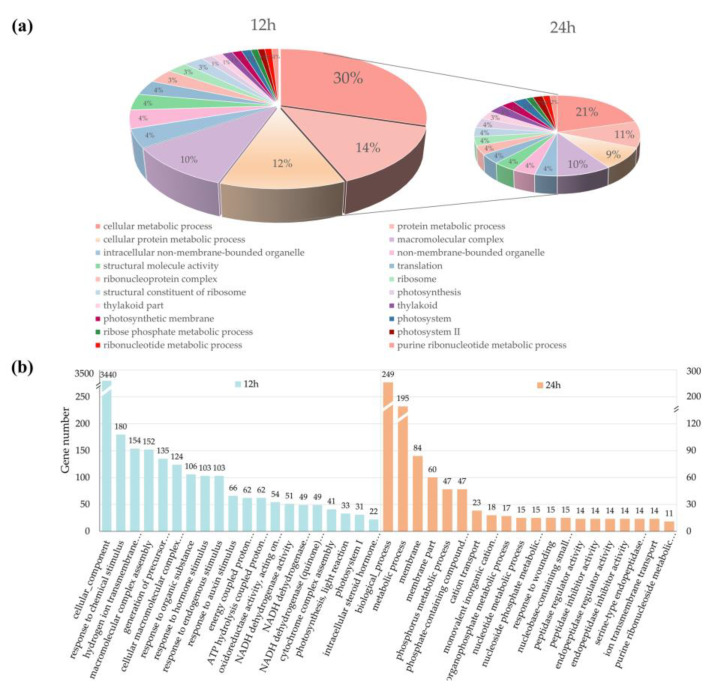
Gene ontology (GO) enrichment analysis of putative target genes (PT genes) neighboring differentially expressed lncRNAs (DE lncRNAs). (**a**) The results of GO analysis based on DE lncRNAs expressed in common at 12 and 24 h. The sector proportion represents the number of PT genes associated with a certain GO category. (**b**) The results of GO analysis based on DE lncRNAs expressed in 12 and 24 h, respectively. Numbers above each bar represent the number of genes in each category.

Previous studies have shown that lncRNAs can regulate the expression of their neighboring genes at the transcriptional level. To reveal potential functional categories of lncRNAs in diverse stages of *F. graminearum* infection, we firstly surveyed 100 kb upstream and downstream of the lncRNAs for predicting PT genes and then performed GO and KEGG enrichment analyses of PT genes neighboring the DE lncRNAs. As a result, a total of 88,748 protein-coding genes were predicted as the PT genes of lncRNAs, which were distributed evenly in all 21 chromosomes of wheat, and the first three chromosomes were 5D (5.47%), 2D (5.46%), and 3B (5.44%). As shown in Figure 4, lncRNAs codifferentially expressed at 12 and 24 h were mainly enriched in the categories of cellular metabolic process, protein metabolic process, macromolecular complex, intracellular nonmembrane-bounded organelle, and translation (Figure 4a). However, stress-stimulus-related genes were only found in wheat 12 hpi, including response to chemical stimulus (180), response to hormone stimulus (103), response to endogenous stimulus (103), and response to auxin stimulus (66). DE lncRNAs of wheat 24 hpi were specifically enriched in peptidase regulator activity (14), peptidase inhibitor activity (14), endopeptidase regulator activity (14), and endopeptidase inhibitor activity (14) (Figure 4b). 

**Figure 5:**
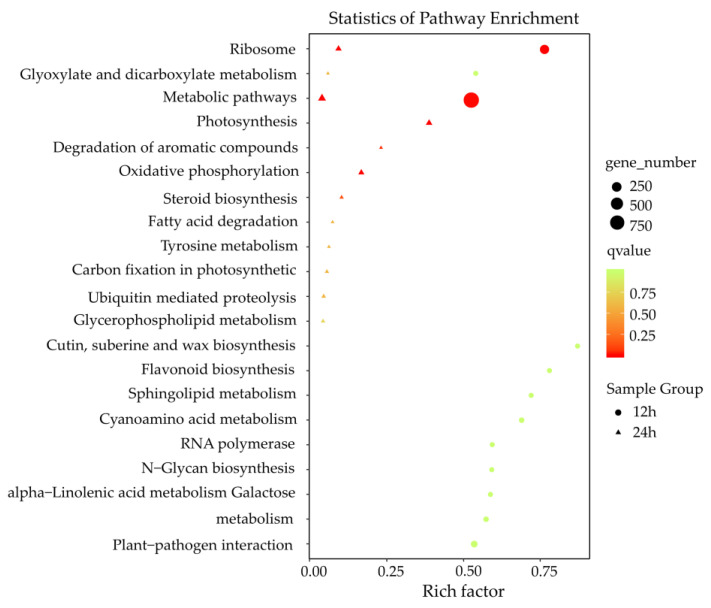
KEGG enrichment analysis of putative target genes (PT genes) neighboring differentially expressed lncRNAs (DE lncRNAs). *X*-axis, the number of PT genes in each category to the ratio of total genes in that category. *Y*-axis, pathway terms. Round-shape, PT genes of the DE lncRNAs 12 h postinoculation. Triangle-shape, PT genes of the DE lncRNAs 24 h postinoculation. The number of genes in each pathway term was reflected by the size of the shapes. The significance of the enrichments is represented by the colors, where red represents a more significant enrichment than green.

The KEGG pathway analysis revealed that disease-resistant pathways were induced against *F. graminearum* infection, such as plant-pathogen interaction, sphingolipid metabolism, cutin/suberin and wax biosynthesis, oxidative phosphorylation, and ubiquitin-mediated proteolysis (Figure 5). Consistent with the results of GO enrichment analysis, pathways related to plant–pathogen interaction, α-linolenic acid metabolism, sphingolipid metabolism, and flavonoid biosynthesis were uniquely induced at 12 h after *F. graminearum-*infection, whereas pathways related to oxidative phosphorylation, ubiquitin-mediated proteolysis, and steroid biosynthesis were mainly induced at 24 h after *F. graminearum* infection. These results suggested that lncRNAs related to various biological process regulations to defend an *F. graminearum* infection had their specific defense models during a certain infection stage.

**Figure 6:**
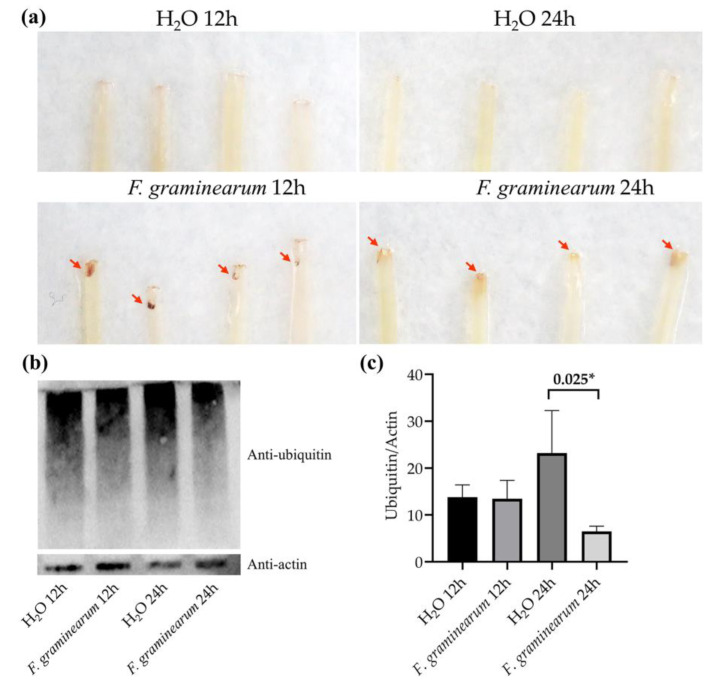
Detection of H_2_O_2_ accumulation and protein ubiquitination of wheat coleoptiles at different stages after inoculation with *F. graminearum*. (**a**) 3,3-Diaminobenzidine (DAB) staining for H_2_O_2_ accumulation. (**b**) Western blotting for protein ubiquitination level. H_2_O 12 h, samples 12 h-inoculated with distilled water; H_2_O 24 h, samples 24 h-inoculated with distilled water; *F. graminearum* 12 h, samples 12 h-inoculated with *F. graminearum*; *F. graminearum* 24 h, samples 24 h-inoculated with *F. graminearum*. (**c**) Gray value analysis of Western blotting results. * *p*  <  0.05.

To test the hypothesis that wheat performed specific patterns against *F. graminearum* infection at different stages, we further detected H_2_O_2_ accumulation and protein ubiquitination at different stages of wheat defense. The coleoptiles infected by *F. graminearum* contained darkly staining tissue in the test of H_2_O_2_ accumulation, indicating that wheat overproduced ROS in response to *F. graminearum* infection. A much heavier stain was present on coleoptiles infected for 12 h, compared with coleoptiles infected for 24 h, indicating the initial 12 h had a stronger defensive response to *F. graminearum* infection (Figure 6a). A protein ubiquitination assay showed that no significant change in the pattern of ubiquitinated proteins was detected in wheat 12 h-inoculated with *F. graminearum*, compared with the control group. Wheat 24 h-inoculated with *F. graminearum*, however, decreased protein ubiquitination compared with the control (Figure 6b,c). The reduced ubiquitination was mainly dispersed with the protein molecular weight range of 100 to 130 kDa. This result was consistent with previous studies that showed that protein ubiquitinations control key aspects of pathogen defense in plants [50,51]. In view of the fact that *Fusarium* mainly colonized in the intercellular space of wheat before 12 h and penetrated the tissue in the following 12 h, we concluded that wheat depends on a hypersensitive response to resist the invasion of *Fusarium* in the first 12 h, and in the following 12 h, relies on protein ubiquitination to resist the proliferation of *Fusarium*.

### 3.4. Candidate lncRNAs Screening for FSB Resistance 

**Figure 7:**
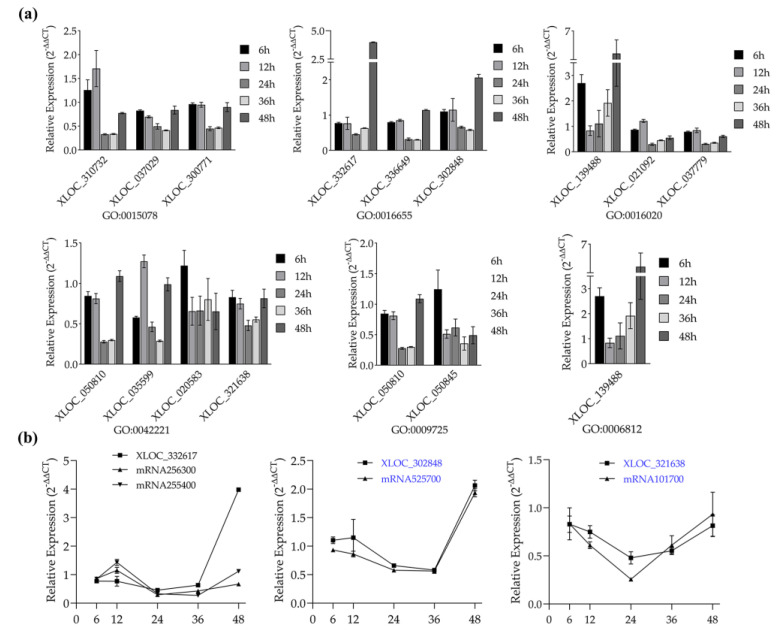
Validation of the defense expression profiles of lncRNAs and expression patterns of lncRNAs coupled with their target genes by using quantitative RT-PCR. (**a**) Defense expression profiles of lncRNAs from six *F. graminearum*-responsive GO terms. Legend, different time points after inoculation with *F. graminearum*. (**b**) Expression pattern of lncRNA-putative target gene pairs after inoculation with *F. graminearum*. *X*-axis, different time points after inoculation with *F. graminearum*. The 2^–ΔΔCT^ method was used to calculate the expression level of lncRNAs and putative target genes. Actin was chosen as the reference gene. Data represent means ± standard deviation (SD) of three replicates.

Knowing that lncRNAs are involved in a variety of wheat defense processes, we further screened specific lncRNAs that function in FSB resistance by evaluating the defense expression profiles of lncRNAs and expression patterns of lncRNAs coupled with their target genes. To ensure the quantitative accuracy of lncRNAs, the lncRNAs that overlapped with mRNAs were excluded by the IGV software. Fourteen DE lncRNAs chosen from six stress response GO terms showed a varying expression pattern over infection time. The lncRNAs of term GO 0009725-response to hormone stimulus significantly decreased from 12 to 36 h, indicating that these lncRNAs function as negative regulators of plant defense for this period. Additionally, the lncRNA XLOC-139488 of term GO 0016020-membrane and term GO 0006812-cation transport was found as a positive regulator of plant defense for 6, 36, and 48 h (Figure 7a). Subsequent expression pattern analysis displayed that some target genes of these DE lncRNAs changed simultaneously in defending *F. graminearum*. As shown in Figure 7b, mRNA525700 and mRNA101700 showed similar trends of expression changes with their paired lncRNA, respectively, both up- or downregulated at a certain stage, implying that these lncRNAs could be potential regulators that function in plant pathogen defense.

### 3.5. Targets Cross-Validated with Refined Meta-QTL 1/Chr. 3B Revealed F. graminearum-Response lncRNAs Act as Potential Regulators of FHB Resistance

**Figure 8:**
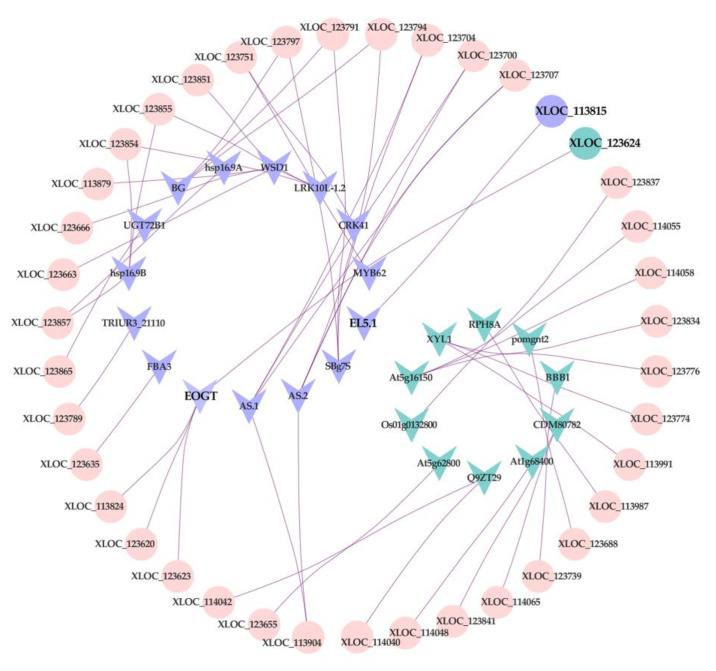
Regulatory network between lncRNAs and putative target genes, cross-validated with the refined meta-QTL 1/chr 3B of FHB resistance. Round-shape: lncRNA. V-shape: putative target gene. Purple represents upregulation, Blue represents downregulation. Pink represents no significant difference.

To explore whether lncRNAs are associated with FHB resistance QTL of wheat, the network of lncRNAs and 324 genes in the meta-QTL 1/Chr. 3B were constructed using *Cytoscape* software analysis (Table S5). Thirty of 324 genes were selected as the candidate genes for cross-validating with lncRNAs, including 16 upregulated and 14 downregulated genes. As shown in Figure 8, 25 genes that matched with 39 lncRNAs were screened as putative target genes, and each of them had one or multiple-matched lncRNA(s), indicating that genes in FHB-resistance QTL may be regulated by lncRNAs to defend *F. graminearum* attacks. Among those 39 lncRNAs, two of them (XLOC_113815, XLOC_123624) were significantly changed after *Fusarium* infection.

## 4. Discussion

An increasing number of studies have recently reported lncRNAs to be essential modulators in a wide range of fundamental biological processes such as regulation of mRNA processing [52,53], activating their neighboring genes using a cis–mediated mechanism [54]. In plants, lncRNAs have been implicated in a wide variety of developmental processes, such as regulation of flowering and vernalization and phosphate homeostasis [55,56]. Moreover, they have been associated with stress responses such as heat stress and pathogen stress [15,57,58]. It has come true that lncRNAs in some plant species can be characterized via sequencing analysis. However, up to now, only a few studies of lncRNAs have been reported in *T. aestivum*, a crucially important food crop, because of its large and complex genome. For example, Zhang et al. identified the expression profiles of lncRNAs in *T. aestivum* under *Puccinia striiformis* and *Blumeria graminis* attack and identified 283 lincRNAs were *P. triiformis*- and *B. graminis*-responsive [29]. Shumayla et al. adopted 52 RNA-seq data and obtained 44,698 lncRNAs during the development and stress response in the *T. aestivum* genome [59]. 

Fusarium head blight, caused mainly by *Fusarium graminearum,* is one of the most important diseases in many wheat-growing areas of the world. In this study, the global expression contour of wheat lncRNAs responding to *F. graminearum* attack at different stages was first established. Through stringent bioinformatic pipelines, a total of 126,391 lncRNAs were reliably discovered. Since clarifying the positional relationship of lncRNAs plays an important role in predicting its function, a more detailed classification was performed. The results showed that the lncRNAs of wheat functioned mainly in the form of lincRNA, which constituted more than 90% of all the lncRNAs. In addition, the structures of lncRNAs were less complex, with the shorter length and fewer exons compared to mRNA transcripts, which made it easier to regulate gene expression.

Comparative analysis was used to evaluate the expression levels of lncRNAs between pathogen stress and natural growth conditions at different stages. A total of 4276 lncRNAs were confirmed to respond to *F. graminearum*. Interestingly, we found that a large number of these DE lncRNAs (over 4130) were specifically expressed at 12 hpi, but only a few (89) were specifically expressed at 24 hpi, indicating the initial stage was the crucial stage for lncRNA-mediated gene regulation of wheat defense against *F. graminearum*. Recent research has shown *F. graminearum* initially colonizes the surface of the wheat coleoptile without immediate penetration, but intercellular space colonization happens at 5–8 h postinoculation [60,61]. Combining these recent results, an initial interaction between wheat and *Fusarium* was proposed. The hyphae colonized the intercellular spaces of the host and triggered the lncRNA-regulated expression of innate immune response genes to cause innate immunity, such as pathogen association molecular pattern (PAMP)-triggered immunity and effector-triggered immunity of the host. During this stage, wheat coleoptile cells were still alive after colonization by *F. graminearum*. The GO and KEGG enrichment analyses revealed that innate immunity stimulated several pathways against *Fusarium*, including plant-pathogen interaction, cutin/suberin and wax biosynthesis, and α-linolenic acid metabolism. Consistent with this, an ROS assay result suggested that wheat defense against *Fusarium* depends on the hypersensitive response in the first 12 h. 

At later proliferation stages (24 hpi), *F. graminearum* produced deoxynivalenol (DON) mycotoxin, which is responsible for the induction of genes encoding proteins involved in ubiquitination and programmed cell death (PCD) [62,63], and the virulence factor fusaoctaxin A, which suppresses host cell defense responses and facilitates *F. graminearum* cell-to-cell hyphal progression [64]. Herein, we found that some *F. graminearum*-responsive lncRNAs identified in this stage were related to the pathway of ubiquitin-mediated proteolysis. Since the ubiquitin–proteasome system (UPS) is heavily implicated in plant immunity [65], we tested the protein ubiquitination of wheat coleoptiles infected by *F. graminearum* to verify the role of UPS in resisting pathogen invasion. Surprisingly, the ubiquitination was reduced in wheat at 24 hpi. Considering the role of DON mycotoxin, we speculate that the host might be able to resist mycotoxin-induced cell death by inducing UPS to regulate some proteins so that they are not degraded in this stage. 

To further validate the novel lncRNAs identified in our study and screen potential regulators resistant to *F. graminearum*, we analyzed the expression patterns between lncRNAs and their putative target genes. Some lncRNA-target gene pairs have already been proven to play important roles in plant defense against bacteria. For example, the lncRNA ALEX1 can activate the jasmonate (JA) pathway against bacterial blight in rice [66]. In this study, we obtained four candidate lncRNAs with high potential in wheat FSB and FHB resistance, which lay a solid foundation for our next research. Due to the wide host range of *Fusarium*, most cereal crops such as wheat and maize lack germplasm resources with high resistance to this pathogen; current protective measures against *Fusarium* mainly rely on fungicides. However, *Fusarium* isolates easily bring out resistant mutations and high-level tolerance to contemporary fungicides because of their genetic characterizations [67]. Therefore, it is necessary to explore new and effective control measures from various aspects to reduce crop diseases caused by *Fusarium*. Previous studies have shown that the pathogenesis in wheat seedlings is similar to that observed in spikes when challenged with FHB pathogens; signal transduction pathways for the activation of the defense genes, such as the cytochrome gene, commonly exist in FHB resistance and FSB resistance. In addition, one wheat cultivar displayed FHB resistance in spikes but exhibited FSB susceptibility in seedlings, or vice versa, which is referred to as resistance inversion [68]. The wheat cultivar (LiangXing 66) used in this study had high FHB susceptibility in spikes; thus, it should be highly resistant to *Fusarium* at the seedling stage. That was conducive to screen the lncRNAs’ functioned in defending *F. graminearum*. Overall, identification of the expression profiles of wheat lncRNAs responding to *Fusarium* not only contributes to fully understand the molecular changes of wheat in defense against *Fusarium* but provides a broader range of selection for candidate factors that play roles in plant immunity, including coding and noncoding RNA. Furthermore, the development of new disease-resistant germplasm resources and new fungicides such as nucleic acid fungicides [69] can be designed for functional lncRNAs, which may have higher biosafety due to their noncoding properties.

In conclusion, lncRNAs could be an important part of regulatory mechanisms of wheat against *F. graminearum* infection. Our study fills the gap on changes of lncRNAs responding to *Fusarium* attack in *T. aestivum* and reveals the interaction between *Fusarium* and wheat. More importantly, we screened several candidate lncRNAs directly associated with plant resistance to pathogens. Given that the function and regulatory mechanism of most of these lncRNAs are still unclear, further research is underway to discover the function of these lncRNAs in *T. aestivum* against *Fusarium* attack at the molecular level.

## Figures and Tables

**Table 1:** Number of major types of lncRNAs.

Types *	Number		
	Sense Strand	Antisense Strand	Total
lncNAT	2471	2561	5032
lincRNA	56,972	56,899	113,871
Intronic	3732	3756	7488

* lncNAT, long noncoding natural antisense transcripts; lincRNA, long intergenic. noncoding RNAs.

## Supplementary Materials

The following are available online at https://www.mdpi.com/2073-4425/11/10/1135/s1. Figure S1: Comparison of genome distribution of DE lncRNAs and mRNAs. Table S1: Stress response GO terms responding to *F. graminearum* attack. Table S2: Primers and sequences used in qRT-PCR analysis. Table S3: Characters of lncRNA and mRNA obtained by RNA-seq. Table S4: Identification of differentially expressed lncRNAs from coleoptiles infected by *F. graminearum*. Table S5: LncRNAs and putative target genes used in network building.
