# Supplementary material for: Genome-Wide Identification and Characterization of Fusarium graminearum-Responsive lncRNAs in Triticum aestivum
Source: Genes (Basel). 2020 Sep 27;11(10):1135. doi: 10.3390/genes11101135 (PMC7601646; doi:10.3390/genes11101135)
Supplement: Supplementary file 1 [file genes-11-01135-s001.zip › genes-927120-supplementary/supplementary/Figure S1-Comparison of genome distribution of DE lncRNAs and mRNAs.docx]

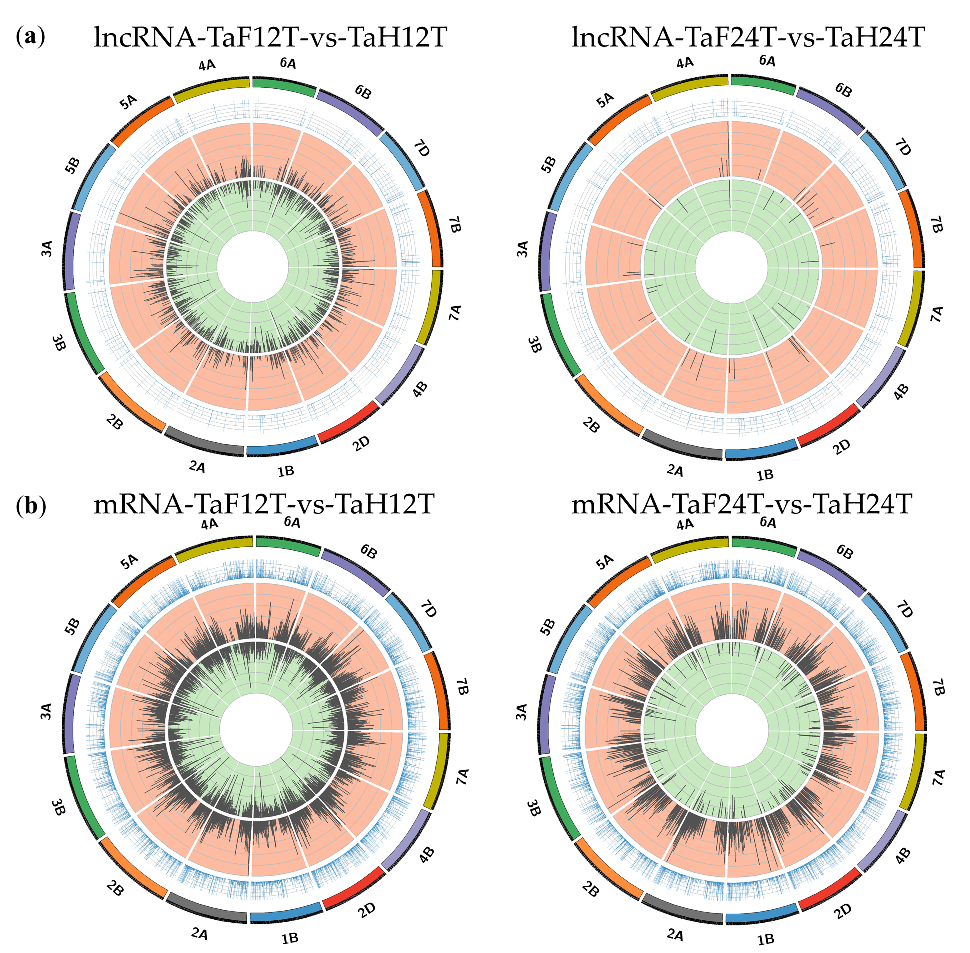


**Figure S1**. Comparison of genome distribution of differently expressed lncRNAs and mRNAs. Outermost circle: chromosome; the second circle, average of the FPKM of this comparative combinations on the corresponding chromosome; the third circle, the distribution of significantly up-regulated lncRNAs/mRNAs on chromosomes (Lines radiate outward from inside of the circle, and the longer the length, the higher the log2 value); the innermost circle is the distribution of significantly down-regulated lncRNAs/mRNAs on chromosomes (Lines radiate inside from outward of the circle, and similarly, the longer the length, the higher the log2 value).
