# Supplementary material for: Genome-Wide Identification and Characterization of Fusarium graminearum-Responsive lncRNAs in Triticum aestivum
Source: Genes (Basel). 2020 Sep 27;11(10):1135. doi: 10.3390/genes11101135 (PMC7601646; doi:10.3390/genes11101135)
Supplement: Supplementary file 1 [file genes-11-01135-s001.zip › genes-927120-supplementary/supplementary/Table S1-Stress responsive GO terms responding to F. asiaticum attack.docx]

Table S1. Stress response GO terms responding to *F. graminearum* attack.

| GO-id | GO-description |
| --- | --- |
| GO:0015078 | hydrogen ion transmembrane transporter activity |
| GO:0009725 | response to hormone stimulus |
| GO:0016655 | oxidoreductase activity, acting on NAD(P)H, quinone or similar compound as acceptor |
| GO:0042221 | response to chemical stimulus |
| GO:0016020 | membrane |
| GO:0006812 | cation transport |
