# Supplementary material for: Genome-Wide Identification and Characterization of Fusarium graminearum-Responsive lncRNAs in Triticum aestivum
Source: Genes (Basel). 2020 Sep 27;11(10):1135. doi: 10.3390/genes11101135 (PMC7601646; doi:10.3390/genes11101135)
Supplement: Supplementary file 1 [file genes-11-01135-s001.zip › genes-927120-supplementary/supplementary/Table S2-Primers and sequences used in qRT-PCR analysis.docx]

Table S2. Primers and sequences used in qRT-PCR analysis.

| Gene | Forward Primer (5' → 3') | Reverse Primer (5' → 3') |
| --- | --- | --- |
| XLOC_310732 | GACAACCTGGCGAACTGAAAC | AACGCTTGTATTGCTCTCCCA |
| XLOC_037029 | TTTTGTCTCATGGTAGCCTGCT | TTGATGATGCCATGTGAATCGC |
| XLOC_300771 | GATCGATTCATCCAAAGCTCGC | AACATCAAAAGTGCGGGTTTCC |
| XLOC_050810 | TTATCACTTATGAGCCGTGCGA | TTATCACTTATGAGCCGTGCGA |
| XLOC_050845 | ATCATCATCTGCAGGCCACAAT | GCAAGAACAGGGAAAAAGGGAC |
| XLOC_332617 | CAAAAGGAGAAATCCGCCCAAG | CAAAAGGAGAAATCCGCCCAAG |
| XLOC_336649 | CCAGTTTGTCTGTCCCCGATTA | CCAGTTTGTCTGTCCCCGATTA |
| XLOC_302848 | CATCCACAGATCCGATCCCAC | CATCCACAGATCCGATCCCAC |
| XLOC_035599 | CTTGGGGTGATCTCGTAGTTCC | CTTGGGGTGATCTCGTAGTTCC |
| XLOC_020583 | GAATAATGCTTGTTGCCTGGCA | GAATAATGCTTGTTGCCTGGCA |
| XLOC_321638 | GCAGCATCAGTCTTGGTCATTC | GCAGCATCAGTCTTGGTCATTC |
| XLOC_139488 | CATTGCCGAGTAGGATTGCTTG | CATTGCCGAGTAGGATTGCTTG |
| XLOC_021092 | CGGAGGAGTATGCGCTTCTAAA | CGGAGGAGTATGCGCTTCTAAA |
| XLOC_037779 | GCCCAACCAGAAACTAGAGCT | GCCCAACCAGAAACTAGAGCT |
| mRNA256300  mRNA255400  mRNA525700  mRNA101700 | CCGTACAAGGAGCTCTAGGAAC  GAAGCTTTACTGTTCCCTGGGA  CGTGTGAAGGCTTGGATTGATG  TGCTGCTCATCCTGTTTCTCAT | GGGACCCGAGAATGCCAATATT  CGAGCTCTTCCAGAGTGGTATC  TCCACAAAGTACACACACCACA  AACTTAAACACAGGGGCAGTCA |
| Actin | CGACTCTGGTGATGGTGTGAG | AGCAAGGTCCAAACGAAGGA |
