# Supplementary figures and images for: Genome-Wide Identification and Characterization of Fusarium graminearum-Responsive lncRNAs in Triticum aestivum
Source: Genes (Basel). 2020 Sep 27;11(10):1135. doi: 10.3390/genes11101135 (PMC7601646; doi:10.3390/genes11101135)

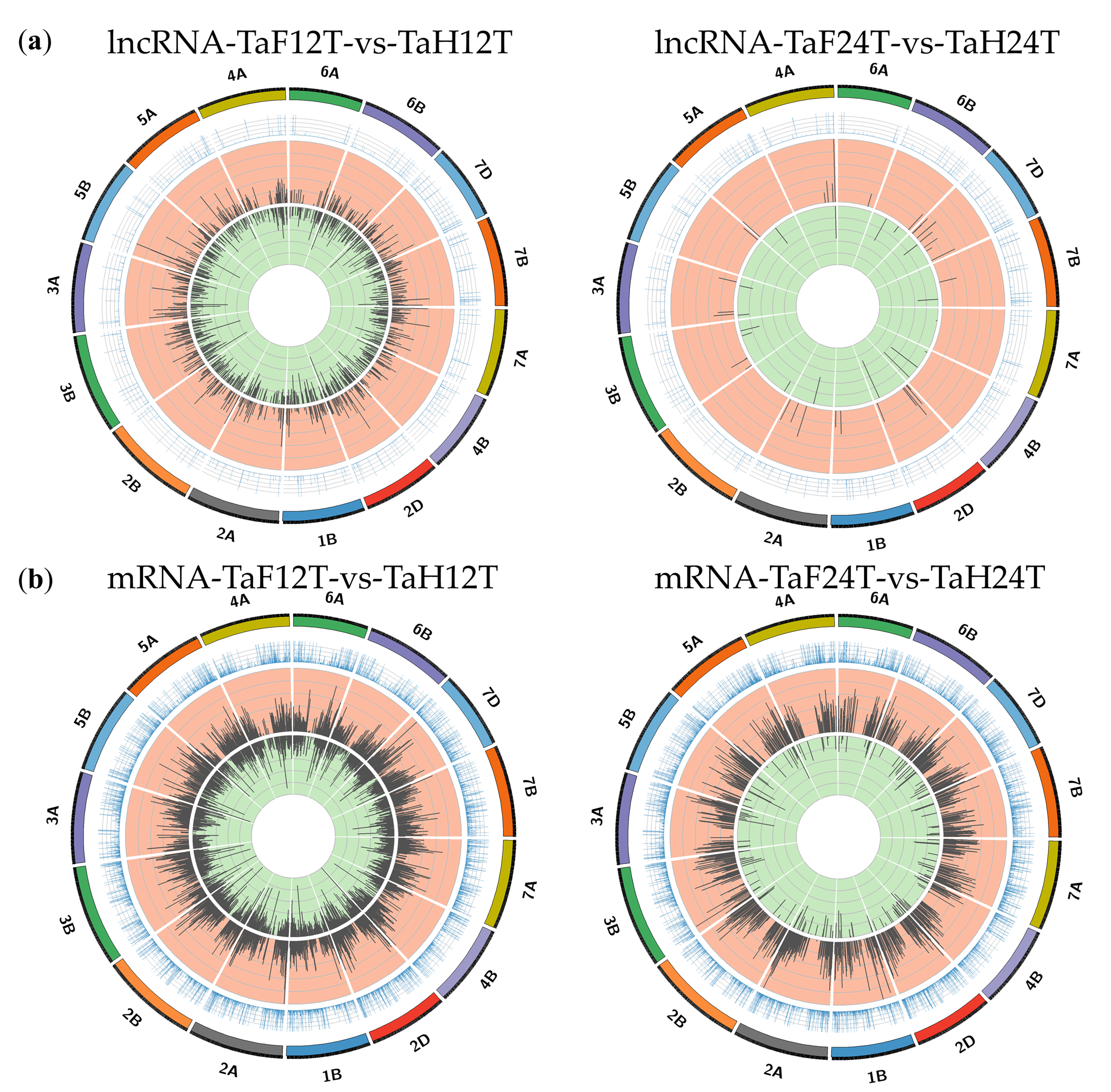

Supplement: Supplementary file 1 [file genes-11-01135-s001.zip › genes-927120-supplementary/supplementary/Figure S1-Comparison of genome distribution of DE lncRNAs and mRNAs.tif]
